# Supplementary material for: Association of ageing-related biomarkers with peripheral neuropathy in colorectal cancer patients up to 2 years after diagnosis
Source: PLoS One. 2025 Sep 26;20(9):e0332579. doi: 10.1371/journal.pone.0332579 (PMC12469108; doi:10.1371/journal.pone.0332579)
Supplement: S1 Information — (DOCX) [file pone.0332579.s001.docx]

**Supplementary**

1. *Relevant information on reporting leukocyte telomere length:*

Based on “*Minimum Reporting Recommendations for PCR-based Telomere Length Measurement”* [2]*,* the following information was given:

Blood was collected via venipuncture in EDTA-containing vials and aliquots of 1 mL were stored at -80 °C until further processing. Maximum storage time was 6 years. DNA was isolated using the QIAamp® DNA Blood Mini Kit (Qiagen) according to the instructions of the manufacturer. DNA quality and concentration were determined using the NanoDrop spectrophotometer (Isogen Life Science, Belgium). The quality of DNA determined as A260/280 ranged between 1.89 and 2.12. DNA was stored at -20 °C until telomere length measurement. q-PCR was performed using 384-multiwells plates (Roche, Switzerland) that were run on a LightCycler 480 machine (Roche). All samples were measured in triplicate, and all samples collected from a single patient were included in the same run. The most outer wells of the plate were not used since we found that PCR results of these wells deviated from those of the more central wells.

For q-PCR the following primers were used [38]:

| Primer name | Sequence |
| --- | --- |
| Telg | ACACTAAGGTTTGGGTTTGGGTTTGGGTTTGGGTTAGTGT |
| Telc | TGTTAGGTATCCCTATCCCTATCCCTATCCCTATCCCTAACA |
| Hbgu | CGGCGGCGGGCGGCGCGGGCTGGGCGGCTTCATCCACGTTCACCTTG |
| Hbgd | GCCCGGCCCGCCGCGCCCGTCCCGCCGGAGGAGAAGTCTGCCGTT |

The wells were filled with 4.6 μL DNA solution and 5.4 μL mastermix, which for each well consisted of 5μL SyBr-green q-PCR master mix (Sensimix SYBR & Fluorescein, GC Biotech) and 0.1μL of each primer (final concentration telg and telc primers was 300nM, final concentration hbgu and hbgd primers was 350nM), making a total volume of 10 μL. The PCR program was as follows: 15’ 95°C enzyme activation; 2 cycles of 15’’ 94°C, 15’’ 49°C; 32 cycles of 15’’ 94°C, 10’’ 62°C, 15’’ 73°C (Ct telomere), 10’’84°C, 15’’ 87°C (Ct b-globulin); 6095°C 0.11°C/s, 5 acquisitions/s (melting curve); 0.05’’ 65°C; 9540°C (cooling). q-PCR signals were processed using the LC480 Conversion program and LinRegPCR (Roche).
