# Supplementary material for: Association of ageing-related biomarkers with peripheral neuropathy in colorectal cancer patients up to 2 years after diagnosis
Source: PLoS One. 2025 Sep 26;20(9):e0332579. doi: 10.1371/journal.pone.0332579 (PMC12469108; doi:10.1371/journal.pone.0332579)
Supplement: S1 Fig — (DOCX) [file pone.0332579.s002.docx]

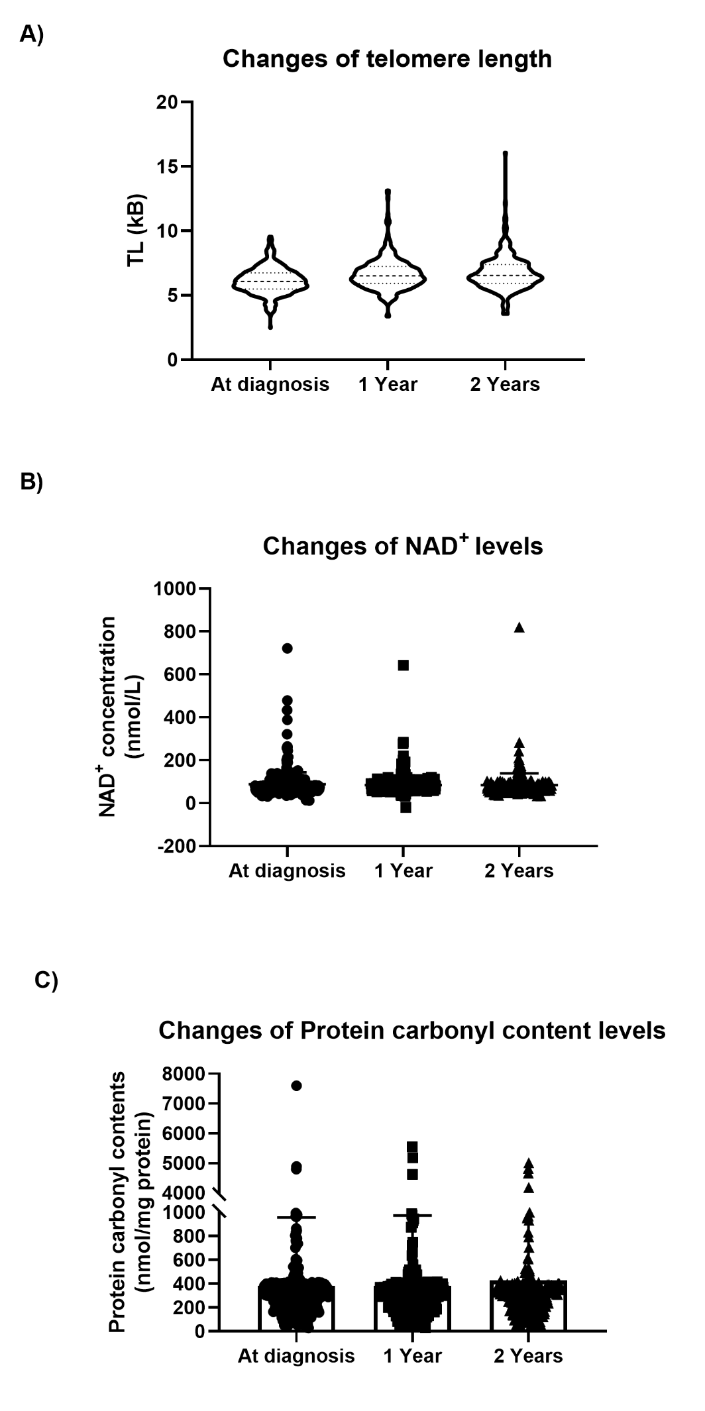


**Figure S1.** Measured biomarkers concentrations of participants at diagnosis, 1-year and 2-year follow up time points. (A) Telomere length (TL, in kB); (B) Plasma NAD^+^ levels; (C) Plasma protein carbonyl contents levels.
