# Supplementary material for: Association of ageing-related biomarkers with peripheral neuropathy in colorectal cancer patients up to 2 years after diagnosis
Source: PLoS One. 2025 Sep 26;20(9):e0332579. doi: 10.1371/journal.pone.0332579 (PMC12469108; doi:10.1371/journal.pone.0332579)
Supplement: S1 Table — (DOCX) [file pone.0332579.s003.docx]

**Table S1**. Telomere length, plasma NAD^+^ levels and protein carbonyl contents levels, and peripheral neuropathy outcomes at post-treatment measurements of participants (data are presented as mean±SD)

|  | At 1-year follow up | At 2-year follow up |
| --- | --- | --- |
| TL (in kB) | 6.7±1.3 | 6.7±1.4 |
| TL (in T/S) | 1.3±0.3 | 1.3±0.4 |
| NAD^+^ (nmol/L) | 84.6±44.6 | 84.7± 55.1 |
| PCC (in nmol/mg protein) | 382.2±590.1 | 428.1±640.9 |
| PN total scores | 24.3±32.3 | 23.1±29.6 |
| SPN | 8.8±14.0 | 8.1±12.2 |
| MPN | 8.1±11.7 | 8.0±11.0 |
| APN | 7.5±14.4 | 7.0±14.2 |
