# Supplementary material for: Association of ageing-related biomarkers with peripheral neuropathy in colorectal cancer patients up to 2 years after diagnosis
Source: PLoS One. 2025 Sep 26;20(9):e0332579. doi: 10.1371/journal.pone.0332579 (PMC12469108; doi:10.1371/journal.pone.0332579)
Supplement: S2 Table — (DOCX) [file pone.0332579.s004.docx]

**Table S2**. Biomarker concentrations of all participants included at diagnosis.

| Biomarkers | No chemotherapy | Receive Chemotherapy | *p*-value |
| --- | --- | --- | --- |
| TL (in kB) | 6.07±1.03 | 6.26±1.04 | 0.09 |
| TL (in T/S) | 1.28±0.28 | 1.24±0.28 | 0.20 |
| NAD^+^ (nmol/L) | 86.96±48.55 | 93.27±64.87 | 0.26 |
| PCC (in nmol/mg protein) | 377.82±525.34 | 389.58±673.19 | 0.42 |
