# Supplementary material for: Association of ageing-related biomarkers with peripheral neuropathy in colorectal cancer patients up to 2 years after diagnosis
Source: PLoS One. 2025 Sep 26;20(9):e0332579. doi: 10.1371/journal.pone.0332579 (PMC12469108; doi:10.1371/journal.pone.0332579)
Supplement: S3 Table — a: Determined by EORTC QLQ-CIPN20 questionnaires. b: model adjusted by age, sex, BMI, receive chemotherapy (yes or no), plasma hemoglobin levels and number of comorbidities. c:Model adjusted by age, sex, BMI, receive chemotherapy (yes or no), and number of comorbidities. (DOCX) [file pone.0332579.s005.docx]

**Table S3**. Sensitivity analysis with and without outliers on the overall longitudinal associations of NAD^+^ and Protein carbonyl content levels with peripheral neuropathy in colorectal cancer survivors followed-up from the time of diagnosis to 2-year post-treatment.

|  | Outliers’ removal | PN total scores^a^  β (95%CI) | SPN^a^  β (95%CI) | MPN^a^  β (95%CI) | APN^a^  β (95%CI) |  |
| --- | --- | --- | --- | --- | --- | --- |
| NAD^+ b^ | Yes | 0.36  (-4.82, 5.53) | -2.28  (-4.30, -0.27**)** | -0.39  (-2.32, 1.55) | 2.73  (-0.00, 5.39) |  |
|  | No | 0.37  (-4.80, 5.54) | -2.29  (-4.31, -0.27) | -0.38  (-2.32, 1.55) | 2.74  (-0.00, 5.40) |  |
| PCC^c^ | Yes | -1.50  (-4.37, 1.42) | -0.70  (-1.72, 0.52) | -0.30  (-1.40, 0.80) | -0.59  (-2.05, 0.95) |  |
|  | No | -1.47  (-4.35, 1.41) | -0.61  (-1.69, 0.46) | -0.29  (-1.39, 0.81) | -0.57  (-2.07, 0.93) |  |

Abbreviations: β, beta-coefficient; CI, confidence interval; PN, peripheral neuropathy; SPN, sensory peripheral neuropathy; MPN, motor peripheral neuropathy; APN, autonomic peripheral neuropathy; PCC, protein carbonyl contents. ^a^: Determined by EORTC QLQ-CIPN20 questionnaires. ^b^: model adjusted by age, sex, BMI, receive chemotherapy (yes or no), plasma hemoglobin levels and number of comorbidities. ^c^:Model adjusted by age, sex, BMI, receive chemotherapy (yes or no), and number of comorbidities.
