# Supplementary material for: Association of ageing-related biomarkers with peripheral neuropathy in colorectal cancer patients up to 2 years after diagnosis
Source: PLoS One. 2025 Sep 26;20(9):e0332579. doi: 10.1371/journal.pone.0332579 (PMC12469108; doi:10.1371/journal.pone.0332579)
Supplement: S4 Table — b: Interaction was tested by introducing an interaction term (either “chemotherapy*TL” or “chemotherapy*NAD+”) into the linear mixed modeling. c: adjusted by age, sex, BMI, chemotherapy (yes/no), plasma hemoglobin levels and number of comorbidities. Statistical significance was denoted in bold. d: β (the beta-coefficient) indicates the overall longitudinal associations in the outcome score. e: β (the beta-coefficient) indicates the intra-individual differences in the outcome scores over time within subjects. f: β (the beta-coefficient) indicates the inter-individual differences in the outcome scores over time between subjects. (DOCX) [file pone.0332579.s006.docx]

**Table S4**. Subgroup analysis based on tumor type (colon or rectal) of longitudinal associations of NAD^+^ and telomere length with peripheral neuropathy in colorectal cancer survivors followed-up from the time of diagnosis to 2-year post-treatment.

|  | TL (in kB)^a^ | | | NAD^+ c^ | | |
| --- | --- | --- | --- | --- | --- | --- |
|  | Tumor type | |  | Tumor type | |  |
|  | Colon | Rectal | P-  _interaction_^b^ | Colon | Rectal | P-_interaction_^b^ |
| *PN* |  |  |  |  |  |  |
| Overall^d^ | 22.48  (9.08, 35.86) | 4.28  (-14.12, 22.68) | 0.131 | -1.22  (-7.50, 5.05) | 4.60  (-4.52,13.73) | 0.524 |
| Intra^e^ | 23.68  (4.51, 42.85) | 6.67  (-15.45, 28.79) | 0.375 | -0.89  (-11.76,9.98) | 19.50  (-7.34,46.35) | 0.196 |
| Inter^f^ | 20.37  (2.89, 37.86) | 4.96  (-23.48, 33.39) | 0.230 | -1.41  (-9.12, 6.30) | 6.89  (-5.54, 19.31) | 0.724 |
| *SPN* |  |  |  |  |  |  |
| Overall^d^ | 7.27  (1.79, 12.75) | 1.77  (-5.90, 9.44) | **0.040** | -2.52  (-5.05, 0.01) | -1.14  (-4.50, 2.23) | 0.982 |
| Intra^e^ | 10.91  (2.35, 19.47) | 2.19  (-7.88, 12.25) | 0.093 | -0.76  (-5.54,4.02) | 9.19  (-3.86,22.23) | 0.738 |
| Inter^f^ | 5.47  (-1.22, 12.16) | 4.14  (-3.86, 12.14) | 0.230 | -3.22  (-6.21,-0.23) | -0.61  (-4.81, 3.58) | 0.788 |
| *MPN* |  |  |  |  |  |  |
| Overall^d^ | 7.37  (2.36, 12.37) | 3.54  (-3.54, 10.62) | 0.681 | -0.80  (-3.14, 1.54) | 0.36  (-3.03,3.77) | 0.507 |
| Intra^e^ | 6.31  (-0.74, 13.36) | 2.16  (-6.55, 10.88) | 0.353 | 0.33  (-3.69,4.35) | 1.45  (-3.58,6.48) | 0.690 |
| Inter^f^ | 7.57  (0.83, 14.31) | 7.85  (-2.55, 18.25) | 0.688 | -1.34  (-4.31, 1.63) | 2.45  (-2.48, 7.38) | 0.185 |
| APN |  |  |  |  |  |  |
| Overall^d^ | 7.61  (0.97, 14.24) | -0.51  (-8.26, 7.24) | 0.273 | 1.62  (-1.58, 4.82) | 5.43  (1.35,9.51) | 0.212 |
| Intra^e^ | 5.69  (-3.44, 14.82) | 2.43  (-6.61, 11.47) | 0.722 | -0.27  (-5.25,4.71) | 6.60  (0.13, 13.08) | **0.024** |
| Inter^f^ | 6.64  (-2.41, 15.70) | -6.88  (-21.57, 7.80) | 0.095 | 2.65  (-1.64, 6.93) | 5.28  (-0.54, 11.09) | 0.799 |

Abbreviations: β, beta-coefficient; CI, confidence interval; PN, peripheral neuropathy; SPN, sensory peripheral neuropathy; MPN, motor peripheral neuropathy; APN, autonomic peripheral neuropathy; TL, telomere length; ^a^: Models were adjusted by age, sex, BMI, chemotherapy (yes/no), and number of comorbidities. ^b^: Interaction was tested by introducing an interaction term (either “chemotherapy*TL” or “chemotherapy*NAD^+^”) into the linear mixed modeling. ^c^: adjusted by age, sex, BMI, chemotherapy (yes/no), plasma hemoglobin levels and number of comorbidities. Statistical significance was denoted in bold. ^d^: β (the beta-coefficient) indicates the overall longitudinal associations in the outcome score. ^e^: β (the beta-coefficient) indicates the intra-individual differences in the outcome scores over time within subjects. ^f^: β (the beta-coefficient) indicates the inter-individual differences in the outcome scores over time between subjects.
