# Supplementary material for: Association of ageing-related biomarkers with peripheral neuropathy in colorectal cancer patients up to 2 years after diagnosis
Source: PLoS One. 2025 Sep 26;20(9):e0332579. doi: 10.1371/journal.pone.0332579 (PMC12469108; doi:10.1371/journal.pone.0332579)
Supplement: S5 Table — Abbreviations: β, beta-coefficient; CI, confidence interval; PN, peripheral neuropathy; SPN, sensory peripheral neuropathy; MPN, motor peripheral neuropathy; APN, autonomic peripheral neuropathy; TL, telomere length; a: Models were adjusted by age, sex, BMI, chemotherapy (yes/no)and number of comorbidities. b: Interaction was tested by introducing an interaction term (either “chemotherapy*TL” or “chemotherapy*NAD+”) into the linear mixed modeling. c: adjusted by age, sex, BMI, chemotherapy (yes/no), plasma hemoglobin levels and number of comorbidities. Statistical significance was denoted in bold. d: β (the beta-coefficient) indicates the overall longitudinal associations in the outcome score. e: β (the beta-coefficient) indicates the intra-individual differences in the outcome scores over time within subjects. f: β (the beta-coefficient) indicates the inter-individual differences in the outcome scores over time between subjects. (DOCX) [file pone.0332579.s007.docx]

**Table S5**. Subgroup analysis based on tumor type (colon or rectal) of longitudinal associations of NAD^+^ and telomere length with peripheral neuropathy in colorectal cancer survivors followed-up from the time of diagnosis to 2-year post-treatment.

|  | TL (in kB)^a^ | | | NAD^+ c^ | | |
| --- | --- | --- | --- | --- | --- | --- |
|  | Age group (68 as a cut-off) | |  | Age group (68 as a cut-off) | |  |
|  | ≤68 | >68 | P-  _interaction_^b^ | ≤68 | >68 | P-_interaction_^b^ |
| *PN* |  |  |  |  |  |  |
| Overall^d^ | 10.16  (-7.16, 27.49) | 18.29  (5.64, 30.93) | 0.634 | -3.72  (-12.09,4.64) | 2.87  (-3.22,8.96) | 0.238 |
| Intra^e^ | 16.08  (-9.76, 41.92) | 18.99  (2.84,35.14) | 0.855 | 7.05  (-9.31,23.42) | 1.11  (-7.42,9.64) | 0.449 |
| Inter^f^ | 12.08  (-8.51, 32.67) | 16.19  (-4.13,36.51) | 0.989 | -5.65  (-15.82,4.52) | 4.71  (-3.97,13.38) | 0.144 |
| *SPN* |  |  |  |  |  |  |
| Overall^d^ | 2.43  (-4.50,9.36) | 6.48  (1.81, 11.15) | 0.377 | -3.30  (-6.60,-0.01) | -1.43  (-3.74,0.89) | 0.416 |
| Intra^e^ | 6.75  (-4.00, 17.50) | 8.02  (1.47,14.56) | 0.745 | 1.91  (-5.10,8.92) | -0.92  (-4.28,2.44) | 0.428 |
| Inter^f^ | 3.69  (-3.95, 11.32) | 6.20  (-1.08,13.49) | 0.913 | -4.18  (-8.14,-0.22) | -1.63  (-4.77,1.52) | 0.332 |
| *MPN* |  |  |  |  |  |  |
| Overall^d^ | 7.19  (0.92, 13.44) | 4.32  (-0.57,9.20) | 0.386 | -0.59  (-3.65,2.47) | -0.52  (-2.77,1.74) | 0.933 |
| Intra^e^ | 6.66  (-2.82,16.15) | 2.74  (-3.26, 8.73) | 0.486 | 2.26  (-3.44,7.97) | 0.52  (-3.12,4.15) | 0.484 |
| Inter^f^ | 8.11  (0.59, 15.64) | 5.61  (-2.35, 13.58) | 0.476 | -0.30  (-4.13,3.52) | -0.41  (-3.88,3.06) | 0.856 |
| APN |  |  |  |  |  |  |
| Overall^d^ | 1.34  (-6.11, 8.80) | 7.00  (-0.76, 14.77) | 0.364 | 0.59  (-3.26,4.44) | 3.60  (-0.01,7.22) | 0.208 |
| Intra^e^ | 3.83  (-6.42,14.08) | 7.85  (-2.56,18.27) | 0.514 | 3.45  (-3.42,10.32) | 1.67  (-3.61,6.96) | 0.643 |
| Inter^f^ | -0.19  (-10.51, 10.12) | 2.93  (-8.29, 14.15) | 0.737 | -1.12  (-5.98,3.73) | 6.42  (1.35,11.48) | **0.036** |

Abbreviations: β, beta-coefficient; CI, confidence interval; PN, peripheral neuropathy; SPN, sensory peripheral neuropathy; MPN, motor peripheral neuropathy; APN, autonomic peripheral neuropathy; TL, telomere length; ^a^: Models were adjusted by age, sex, BMI, chemotherapy (yes/no)and number of comorbidities. ^b^: Interaction was tested by introducing an interaction term (either “chemotherapy*TL” or “chemotherapy*NAD^+^”) into the linear mixed modeling. ^c^: adjusted by age, sex, BMI, chemotherapy (yes/no), plasma hemoglobin levels and number of comorbidities. Statistical significance was denoted in bold. ^d^: β (the beta-coefficient) indicates the overall longitudinal associations in the outcome score. ^e^: β (the beta-coefficient) indicates the intra-individual differences in the outcome scores over time within subjects. ^f^: β (the beta-coefficient) indicates the inter-individual differences in the outcome scores over time between subjects.
