# Supplementary material for: Association of ageing-related biomarkers with peripheral neuropathy in colorectal cancer patients up to 2 years after diagnosis
Source: PLoS One. 2025 Sep 26;20(9):e0332579. doi: 10.1371/journal.pone.0332579 (PMC12469108; doi:10.1371/journal.pone.0332579)
Supplement: S6 Table — Abbreviations: β, beta-coefficient; CI, confidence interval; PN, peripheral neuropathy; SPN, sensory peripheral neuropathy; MPN, motor peripheral neuropathy; APN, autonomic peripheral neuropathy; TL, telomere length;a: Models were adjusted by age, sex, BMI, chemotherapy (yes/no) and number of comorbidities. b: Interaction was tested by introducing an interaction term (either “chemotherapy*TL” or “chemotherapy*NAD+”) into the linear mixed modeling. c: adjusted by age, sex, BMI, plasma hemoglobin levels, chemotherapy (yes/no) and number of comorbidities. Statistical significance was denoted in bold. d: β (the beta-coefficient) indicates the overall longitudinal associations in the outcome score. e: β (the beta-coefficient) indicates the intra-individual differences in the outcome scores over time within subjects. f: β (the beta-coefficient) indicates the inter-individual differences in the outcome scores over time between subjects. (DOCX) [file pone.0332579.s008.docx]

**Table S6**. The overall, intra- and inter-individual longitudinal associations of telomere length and plasma NAD^+^ levels with peripheral neuropathy stratified by moderate-to-vigorous intensity physical activity (MVPA) at diagnosis in colorectal cancer survivors.

|  | TL (in kB)^a^ | | | NAD^+ c^ | | |
| --- | --- | --- | --- | --- | --- | --- |
|  | MVPA (hours/week) | |  | MVPA (hours/week) | |  |
|  | ≤11 | >11 | P-  _interaction_^b^ | ≤11 | >11 | P-_interaction_^b^ |
| *PN* |  |  |  |  |  |  |
| Overall^d^ | 20.94  (2.80,39.09) | 15.82  (2.60,29.05) | 0.658 | -0.35  (-7.76,7.06) | -1.08  (-8.51,6.34) | 0.804 |
| Intra^e^ | 21.04  (-3.53, 45.61) | 18.23  (-0.22,36.69) | 0.712 | 2.51  (-9.37,14.40) | 6.06  (-7.49,19.60) | 0.557 |
| Inter^f^ | 20.00  (-7.34,47.34) | 13.22  (-2.61,29.04) | 0.676 | 0.19  (-9.52,9.90) | -4.31  (-13.34,4.72) | 0.720 |
| *SPN* |  |  |  |  |  |  |
| Overall^d^ | 5.00  (-2.30,12.31) | 6.68  (1.16,12.20) | 0.767 | -2.55  (-5.38,0.28) | -1.60  (-4.72,1.51) | 0.622 |
| Intra^e^ | 8.50  (-2.53,19.53) | 8.28  (-0.53,17.09) | 0.648 | -0.34  (-5.14,4.46) | 2.35  (-3.63,8.32) | 0.455 |
| Inter^f^ | 5.09  (-4.37,14.55) | 4.71  (-1.51, 10.93) | 0.993 | -2.73  (-6.31, 0.85) | -3.19  (-6.89,0.52) | 0.832 |
| *MPN* |  |  |  |  |  |  |
| Overall^d^ | 10.16  (3.41,16.90) | 3.98  (-0.84, 8.80) | 0.133 | -0.60  (-3.33, 2.13) | -1.30  (-3.91,1.31) | 0.857 |
| Intra^e^ | 9.19  (-0.13, 18.51) | 3.14  (-3.63,9.91) | 0.306 | 1.74  (-2.34,5.82) | -0.85  (-5.62,3.92) | 0.586 |
| Inter^f^ | 10.07  (-0.06, 20.20) | 5.02  (-0.54,10.58) | 0.265 | -1.04  (-4.82,2.75) | -1.55  (-4.72,1.63) | 0.834 |
| APN |  |  |  |  |  |  |
| Overall^d^ | 4.46  (-4.45, 13.38) | 4.71  (-2.17,11.58) | 0.798 | 2.69  (-1.09,6.47) | 1.63  (-2.32,5.58) | 0.939 |
| Intra^e^ | 5.60  (-6.52, 17.72) | 4.79  (-4.87,14.44) | 0.954 | 0.83  (-5.44,7.10) | 4.26  (-2.51,11.02) | 0.409 |
| Inter^f^ | 3.57  (-9.63,16.77) | 2.94  (-6.02, 11.89) | 0.832 | 3.95  (-0.86,8.75) | 0.30  (-4.66,5.25) | 0.546 |

Abbreviations: β, beta-coefficient; CI, confidence interval; PN, peripheral neuropathy; SPN, sensory peripheral neuropathy; MPN, motor peripheral neuropathy; APN, autonomic peripheral neuropathy; TL, telomere length; ^a^: Models were adjusted by age, sex, BMI, chemotherapy (yes/no) and number of comorbidities. ^b^: Interaction was tested by introducing an interaction term (either “chemotherapy*TL” or “chemotherapy*NAD^+^”) into the linear mixed modeling. ^c^: adjusted by age, sex, BMI, plasma hemoglobin levels, chemotherapy (yes/no) and number of comorbidities. Statistical significance was denoted in bold. ^d^: β (the beta-coefficient) indicates the overall longitudinal associations in the outcome score. ^e^: β (the beta-coefficient) indicates the intra-individual differences in the outcome scores over time within subjects. ^f^: β (the beta-coefficient) indicates the inter-individual differences in the outcome scores over time between subjects.
