# Supplementary material for: Association of ageing-related biomarkers with peripheral neuropathy in colorectal cancer patients up to 2 years after diagnosis
Source: PLoS One. 2025 Sep 26;20(9):e0332579. doi: 10.1371/journal.pone.0332579 (PMC12469108; doi:10.1371/journal.pone.0332579)
Supplement: Attachment — Submitted filename: R2_Response to reviewers.docx [file pone.0332579.s009.docx]

PONE-D-25-13325R1

Dear Editor (and Reviewers),

We thank you for your thoughtful and constructive comments. Below we provide a point-by-point response. Each comment is restated in regular font, followed by our response in *italics*, and a suggested manuscript revision in **red**, including the page number and lines corresponding to the manuscript with track changes. We hope the changes we have made based on your feedback improve the clarity, quality, and impact of our manuscript and will now be suitable for publication in PLOS ONE.

--

**Additional Editor Comments:**

Thanks for submitting your revised paper to PLOS ONE. The previous reviewers are not available this time. Thus, I have carefully assessed your revision work.

*Thank you for taking the time to review our work and your insightful comments.*

Based on the manuscript revisions, here are critical questions for the authors to address:

1. NAD⁺ Measurement Validity in Plasma: The manuscript states plasma NAD⁺ was measured due to "multicenter design and logistical constraints," citing Lautrup et al. (2024) to justify its relevance to systemic NAD⁺ status. However, Lautrup’s review primarily discusses intracellular NAD⁺ roles. Please provide additional evidence that plasma NAD⁺ levels reliably reflect tissue-level NAD⁺ bioavailability in neurons, especially given that intracellular NAD⁺ is more directly linked to neuroprotection. Did you validate this against cellular NAD⁺ in a subset of samples?

*Thank you for your critical remarks, the paper of Lautrup et al. (2024) was chosen since this is an extensive review of all aspects of NAD^+^, including the outcome of studies that determined the plasma NAD^+^ metabolome as a marker of tissue NAD^+^ metabolome (for example the paper by Clement et al. 2019 (Clement J, Wong M, Poljak A, Sachdev P, Braidy N. The Plasma NAD+ Metabolome Is Dysregulated in "Normal" Aging. Rejuvenation Res. 2019 Apr;22(2):121-130. doi: 10.1089/rej.2018.2077. Epub 2018 Oct 23).*

*Although there are no human data correlating plasma NAD with brain NAD, recently in a mouse model a positive correlation between plasma and brain levels of NAD was observed by Ishima et al. (2024) (Ishima T, Kimura N, Kobayashi M, Nagai R, Osaka H, Aizawa, K. A Simple, Fast, Sensitive LC-MS/MS Method to Quantify NAD(H) in Biological Samples: Plasma NAD(H) Measurement to Monitor Brain Pathophysiology. Int J Mol Sci. 2024 Feb 15;25(4):2325. doi: 10.3390/ijms25042325). In our study it was not possible to measure NAD^+^ in (specific) white blood cell populations and plasma of the same patient since it was not possible to process blood for isolation of (specific) white blood cells immediately after sampling.*

*In our study, plasma NAD is used as a marker of the NAD^+^-status of other tissues of a patient, not specifically neuronal or blood cells. The method used is an enzymatic cycling assay and determines total NAD (NAD^+^ and NADH). The rationale is that an increase in NAD^+^-consuming enzyme activities associated with chronic (neuro)-inflammation, including CD38, PARP-1 and possibly SARM1 would lead to a decreased NAD^+^-status.*

*In the manuscript the text and references are adapted on page 5 in lines 112-118:*

Thawed plasma samples were centrifuged to use only soluble parts for biomarker measurements. Given the multicenter design and logistical constraints, we measured NAD in plasma rather than in cellular fractions. Plasma total NAD levels (NAD^+^ and NADH), which have been shown to decline with age and reflect systemic NAD⁺ status (37, 38), were quantified using a sensitive enzymatic cycling assay adapted from Bernofsky & Swan as described previously (39). To control for potential hemolysis plasma free hemoglobin (Hb) was also measured in triplicate (40-41).

2. Inconsistency in LTL-PN Association: Longer LTL was unexpectedly associated with worse PN (Table 2). The discussion proposes chemotherapy may select for hematopoietic stem cells with longer telomeres (Benitez-Buelga et al. 2015), but this does not explain why longer LTL correlates with neuropathy severity. Could residual confounding (e.g., unaccounted inflammatory markers or cell-type composition shifts) drive this association? Provide sensitivity analyses adjusting for NLR (neutrophil-lymphocyte ratio) or other immune indices.

*In the discussion we proposed that a shift in leucocyte populations may underlie the observed changes in LTL page 10, lines 293-300. However, due to logistic obstacles it was not possible to isolate (specific) white blood cell populations immediately after blood sampling to determine the cell-type composition of blood samples. This is indeed an important aspect worth a more elaborate discussion which is now added to page 10.*

We measured LTL instead of tissue TL as it is less invasive, but its extrapolation to neuronal tissue is uncertain (70). LTL is largely dependent on leukocyte turnover, influenced by chronic inflammation, oxidative stress and shifts in specific leukocyte populations (71). Moreover, telomeres of hematopoietic stem cells may also be damaged during chemotherapy, resulting in an increased shortening of telomeres (75). Cells with critically shortened telomeres will become apoptotic or senescent, and no longer contribute to the circulating leukocyte population. This results in a selection for stem cells with longer telomeres, and an increase in average LTL in the years following treatment (76). Therapy-induced damage to non-cancer tissue may result in permanent damage or senescence in non-proliferating cells such as neurons, which may underlie NP symptoms. Hence, a selection for cells with longer telomeres in proliferating cells such as hematopoietic stem cells may be associated with permanent damage in non-proliferating neuronal cells, which may explain the observed association.

3. Data Availability Limitations: Data are accessible via PROFILES registry "upon written request and possible purchase." PLOS ONE mandates unrestricted public access. Clarify how this complies with PLOS’s data policy. Will data be deposited in a public repository (e.g., Dryad) with anonymized identifiers to ensure accessibility without barriers?

*The data supporting the findings of our study are available free of charge for non-commercial research purposes. Approval of the storage and sharing of these data with third parties has been granted by the METC as part of the ethics approval for our research. Researchers can request access via the PROFILES registry (*[*www.profilesregistry.nl*](http://www.profilesregistry.nl)*), and all reasonable requests for scientific purposes will be honored at no cost.*

*We do not deposit the data publicly due to legal and ethical considerations related to participant privacy, as outlined by the ethics board. However, data can be provided under a data-sharing agreement to ensure compliance with these requirements. Please let us know if you need any additional information.*

4. Chemotherapy Subgroup Heterogeneity: Table 3 shows strong NAD⁺-PN associations in chemotherapy-treated patients but a positive trend (β=3.75, p=NS) in non-chemotherapy patients. Is this divergence biologically plausible? Could unmeasured factors (e.g., specific neurotoxic agents like oxaliplatin) explain this? Provide subgroup analysis by chemotherapy regimen if feasible.

*We agree that this would be a valuable addition to the manuscript. However, unfortunately this detailed information is unavailable. We state this as a limitation in the manuscript on page 11, lines 314-316.*

Furthermore detailed data on the type and duration of chemotherapy regimens is unavailable, preventing stratified analyses by specific agents such as oxaliplatin, known to be highly neurotoxic.

5. Protein Carbonyl (PCC) Stability Concerns: PCC showed no association with PN. The authors note samples were stored >6 years, and stability beyond 1 month at −70°C is unverified (Firuzi et al. 2006). Provide evidence that PCC remained stable under your storage conditions (e.g., correlation between baseline/follow-up levels in controls or spike-in experiments).

*In the discussion (page 10 lines 281-285 we currently state:*

Another factor could be the stability of plasma samples during storage. The oldest samples have been stored for more than 6 years before measurement, and although PCC are considered relatively stable, and were reported to be not changed after one month of storage at -70°C (67), it remains to be determined whether plasma PCC is stable after storage for several years.

*Determination of the stability of PCCs during storage at -70°C would have required taking a control sample at the start of inclusion in 2016, immediately measuring PCCs, and storing aliquots of the plasma for measurements more than six years later. This would by definition introduce inter-assay variability making it difficult to assess the real stability of PCCs during storage of plasma for over six years. Moreover, in 2016 the measurement of PCCs was not operational making such a scenario impossible. PCC measurement was selected at a later stage based on the information that it is regarded as one of the most stable biomarkers of oxidative stress.*

*In the limitations of the study, potential problems with stability of biomarkers such as PCCs and NAD+ after prolonged storage of plasma at -70°C are now included on page 11 in lines 307-311.*

Due to the multicenter study design, an inclusion period of a little more than three years, and a follow-up of two years, some blood and plasma samples were stored for a period of a little more than six years before analyses were done. Since the stability of PCCs and NAD+ during prolonged storage of plasma at -70°C is not known, this is a limitation of this study.

6. Attrition Bias in Longitudinal Data: Only 37% completed the 2-year follow-up (page 18), with responders likely healthier than non-responders. How was attrition addressed in mixed models? Provide a comparison of baseline characteristics between completers vs. dropouts to quantify potential bias.

*We acknowledge that declining response rates over time (from 64% at baseline to 37% at 2-year follow-up) may introduce attrition bias. Given the number of outcome variables and predictors assessed in our study, the exact number of participants included varies per analysis, which complicates a uniform attrition analysis. However, it is plausible that patients with more advanced disease stages or greater comorbidity were more likely to drop out due to illness or death. This selective attrition could potentially attenuate the observed associations, particularly if those participants had higher oxidative stress levels, shorter telomere lengths, and greater neuropathy burden. Therefore, our findings might represent a conservative estimate of the true associations. We now explicitly acknowledge this potential bias in the discussion on page 11 lines 301-307.*

Study limitations include declining response rates across follow-up waves, which may have introduced attrition bias—particularly if participants with more advanced disease or comorbidities were more likely to drop out due to illness or death—potentially leading to an underestimation of the observed associations between biological aging markers, oxidative stress, and peripheral neuropathy.

*In addition, we report the results from the comparison between completers vs dropouts quantifying potential bias on page 7, lines 186-188.*

Previously published details [5,49] show that respondents were younger, more often male, and more likely to have received chemotherapy than non-respondents. At diagnosis, 61.5% of participating CRC patients were male, with a mean age of 67.7 years (SD 8.9), with 31.7% receiving chemotherapy, Table 1.

7. Ethical Oversight Clarification: The ethics statement lists approval number "NLS1119.060.14" (page 4), but the Methods section (page 44) cites "NL51119.060.14." Please confirm the correct approval number and ensure consistency throughout the manuscript.

*Thank you for pointing out this error. The fault has been corrected in the manuscript on page 4 in accordance with the number on page 44. That is, "NL51119.060.14"*

8. I did not see the detailed responses to reviewers' comments, and please provide them just in the cover letter for me to find them.

*We are sorry for the inconvenience of you not receiving the rebuttal letter (it was attached to our revised submission on pages 62-77). We want to thank you very much for your efforts to read our manuscript without them. Of course we now provide our rebuttal in the ‘cover letter’ section of the submission system.*
